# Fast-track surgery and telerehabilitation protocol in unicompartmental knee arthroplasty leads to superior outcomes when compared with the standard protocol: a propensity-matched pilot study

**DOI:** 10.1186/s43019-022-00173-z

**Published:** 2022-12-12

**Authors:** Luca De Berardinis, Marco Senarighi, Carlo Ciccullo, Fabiana Forte, Marco Spezia, Antonio Pompilio Gigante

**Affiliations:** 1 Clinical Orthopedics, Department of Clinical and Molecular Science, School of Medicine, Università Politecnica delle Marche, Via Tronto, 10/a, 60126 Ancona, AN Italy; 2 Specialist of Physical Medicine & Rehabilitation, COQ (Centro Ortopedico di Quadrante), Madonna del Popolo Hospital, Via Lungolago Buozzi 25, 28887 Omegna, VB Italy; 3 Surgical Director of Department of Orthopaedics, COQ (Centro Ortopedico di Quadrante), Madonna del Popolo Hospital, Via Lungolago Buozzi 25, 28887 Omegna, VB Italy

**Keywords:** Unicompartmental knee arthroplasty, Knee arthroplasty, Fast-track program, Home rehabilitation, Telerehabilitation, Enhanced recovery after surgery

## Abstract

**Background:**

Several strategies have been devised to reduce the length of stay after orthopedic surgery. Telerehabilitation has proved effective in functional outcomes after orthopedic procedures and is appreciated by patients. There is limited information on fast-track surgery and telerehabilitation protocols for unicompartmental knee arthroplasty (UKA). The purpose of this pilot study was to report and compare functional outcomes and satisfaction levels during first 12 months of recovery in patients who underwent UKA according to a fast-track and telerehabilitation protocol (G1) or standard surgery and rehabilitation program (G2).

**Methods:**

Data were retrospectively collected and reviewed for all elective UKAs from January 2018 to November 2019. A total of seven patients undergoing UKA according to the fast-track and telerehabilitation protocol were propensity score matched (1:3 ratio) to 21 patients undergoing standard surgery and rehabilitation. Patients were matched for age, sex, body mass index (BMI), and laterality. The Western Ontario and McMaster University (WOMAC) osteoarthritis index and range of motion (ROM) were collected pre- and postoperatively in both groups for 12 months. In addition, patient’ satisfaction was collected at 40 days.

**Results:**

The G1 group demonstrated significantly better outcomes in WOMAC index scores at 2, 15, and 40 days (*p* < 0.001, *p* < 0.001, *p* < 0.020, respectively) and a significantly greater knee ROM after surgery and at 2, 15, 40, and 12 months (*p* < 0.001, *p* < 0.001, *p* = 0.014, *p* < 0.001, *p* = 0.003, respectively). No patients in either group had postoperative complications. One patient was not completely satisfied in the G2, while no one in G1 reported not being completely satisfied (*p* = 1.000).

**Conclusions:**

This fast-track and telerehabilitation protocol after UKA can potentially be applied to patients as it is safe and effective. At 12-months follow-up, both groups reported favorable outcomes after UKA. However, the G1 score was better regarding WOMAC and ROM when compared with the propensity score-matched G2 program. A larger study is warranted to explore the role of fast-track and telerehabilitation in clinical and functional outcomes of UKA.

## Introduction

In the last 20 years, isolated unicompartmental osteoarthritis has increasingly been treated by unicompartmental knee arthroplasty (UKA), which has a faster short-term recovery and reduced postoperative pain and morbidity than total knee arthroplasty [1, 2]. In the past decade, fast-track protocols have been introduced in clinical and care pathways for elective joint replacement [3, 4]. They are characterized by changes to the surgical procedure and the post-discharge function [5, 6] and by the participation of different specialists at all stages [3, 7]. Their goal is to reduce the physiological and psychological stress of surgery, achieve earlier mobilization and faster recovery, and reduce length of stay (LOS) and hospital costs [8].

Notably, changes in perioperative analgesia, nursing care, and rehabilitation have markedly reduced LOS for these procedures; in particular, effective pain management is critical for faster recovery and earlier discharge after lower limb arthroplasty [9, 10]. Other key factors for successful recovery are preoperative rehabilitation and muscle strengthening as well as early postoperative rehabilitation [11, 12]. Interestingly, shorter hospital stays are associated with better total joint replacement outcomes and greater patient satisfaction [13, 14]. Yet, UKA is still considered a major procedure requiring prolonged hospitalization, mainly due to surgeons’ concerns over postoperative complications, such as an increase in the rates of readmission or return to theater, postoperative blood transfusion requirements, cardiac ischemic events, and 30- and 90-day mortality, as well as patients’ concerns over pain control and rehabilitation at home [15].

In recent years, telerehabilitation has increasingly been used as a supplement or even an alternative to conventional face-to-face physical therapy [16]. In telerehabilitation, sensors and software enable therapists to assess progress remotely, and patients feel closely monitored and supported [17]. Results are comparable to those obtained with outpatient physical therapy and even with face-to-face home rehabilitation [18]. Moreover, telerehabilitation is well accepted by patients [19, 20].

To the best of our knowledge, this is the first study that use a propensity score-matched analysis to compare clinical outcomes and satisfaction rate in patients who underwent UKA following a fast-track and telerehabilitation protocol (G1) or a standard surgery and rehabilitation protocol (G2).

The purpose of this pilot study was to report and compare functional outcomes [i.e., Western Ontario and McMaster University (WOMAC) and range of motion (ROM)], complications, and satisfaction levels during the first 12 months of recovery in patients who underwent UKA according to protocol G1 or program G2. We hypothesized that patients who underwent a fast-track and telerehabilitation protocol could achieve earlier better functional outcomes and satisfaction levels without a higher complication rate.

## Methods

### Study design:

This was a pilot retrospective clinical trial.

### Patient selection criteria

**Fig. 1:**
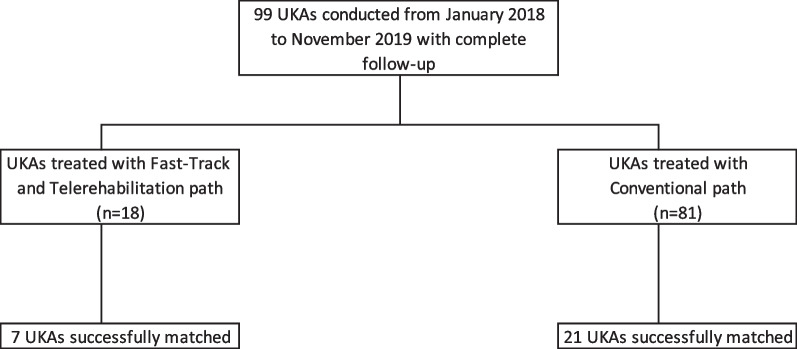
Flow of patients during the study period. *UKA* unicompartmental knee arthroplasty

The data were retrospectively collected and reviewed for all patients who underwent primary UKA by the senior author (M.S.). Patients were included if they underwent a UKA in a fast-track surgery and telerehabilitation protocol (Group 1) or in a standard protocol (Group 2). Patients in Group 1 were enrolled according to fast-track and telerehabilitation protocol inclusion criteria. From January 2018 to November 2019, a total of 99 patients underwent UKA. Of these,18 followed the fast-track and telerehabilitation protocol, while 81 followed the standard protocol, 3 of which failed to complete the 12-month follow-up. After propensity score matching (PSM), 7 patients of Group 1 were successfully matched with 21 patients of Group 2 (Fig. 1). There were no statistically significant differences in body mass index (BMI), smoking status, alcohol use, and allergies between G1 and G2 (Table 1).

**Table 1:** Preoperative and perioperative characteristics of the UKA patients

	Pre match			Post match		
Variable	Fast-track + telerehabilitation protocol	Standard protocol	*p*-Value	Fast-track + telerehabilitation protocol	Standard protocol	*p*-Value
Age in years, mean (SD) [range]	65.0 (117) [48.0–76.0]	69.4 (11.1) [42.0–86.0]	0.164	61.6 (12.3) [48.0–76.0]	64.6 (14.1) [42.0–83.0]	0.490
Gender						
Male (%)	15 (83.3)	21 (26.9)	< 0.001	5 (71.4)	15 (71.4)	1.000
Female (%)	3 (16.7)	57.00 (73.1)		2 (28.6)	6 (28.6)	
Side						
Right (%)	12 (66.7)	36 (46.2)	0.19	4 (57.1)	12 (57.1)	1.000
Left (%)	6 (33.3)	42 (53.8)		3 (42.9)	9 (42.9)	
Localization of osteoarthritis:						
Medial (%)	18 (100)	72 (92.3)	0.59	7 (100)	18 (85.7)	0.551
Lateral (%)	0 (0)	6 (7.7)		0 (0)	3 (14.3)	
BMI (kg/m^2^), mean (SD) [range]	26.8 (2.3) [23.0–29.0]	28.2 (4.1) [21.0–38.4]	0.555	27.4 (2.2) [23.0–29.0]	26.9 (2.8) [22.3–31.4]	0.490
Smoking status:						
Never (%)	9 (50)	54 (69.2)	0.001	4 (57.1)	9 (42.9)	0.394
Current smoker (%)	9 (50)	9 (11.6)		3 (42.9)	6 (28.6)	
Former (%)	0 (0)	15 (19.2)		0 (0)	6 (28.6)	
Alcohol						
Never (%)	3 (16.767)	30 (38.5)	0.129	1 (14.3)	6 (28.6)	0.709
With meals (%)	12 (66.7)	42 (53.8)		4 (57.1)	12 (57.1)	
During the day (%)	3 (16.7)	6 (7.7)		2 (28.6)	3 (14.3)	
ASA class (%)						
ASA 1	6 (33.3)	16 (20.6)	0.088	3 (42.9)	6 (28.6)	0.394
ASA 2	12 (66.7)	48 (61.5)		4 (57.1)	9 (42.9)	
ASA 3	0	14 (17.9)		0	6 (28.6)	
Allergies						
Yes (%)	3 (16.7)	12 (15.4)	1.000	0 (0)	3 (14.3)	0.551
No (%)	15 (83.3)	66 (84.6)		7 (100)	18 (85.7)	
Operation time (min), mean (SD) [range]	45.0 (4.2) [40.0–50.0]	48.3 (16.2) [25.0–100.0]	0.803	45.7 (4.5) [40.0–50.0]	55.0 (15.0) [35.0–75.0]	0.184
Drain						
Yes (%)	0 (0)	6 (7.7)	0.59	0 (0)	3 (14.3)	0.551
No (%)	18 (100)	72 (92.3)		7 (100)	18 (85.7)	
Length of hospital stay (days), mean (SD) [range]	3.17 (0.4) [3.0–4.0]	13.2 (1.8) [10.0–16.0]	< 0.001	3.3 (0.5) [3.0–4.0]	13.0 (1.9) [10.0–15.0]	< 0.001

*UKA*, unicompartmental knee arthroplasty; *SD*, standard deviation; *BMI*, body mass index; *ASA*, American Society of Anesthesiologists

The minimum follow-up time was 12 months and included Western Ontario and McMaster University (WOMAC) osteoarthritis index, range of motion (ROM), and patient satisfaction. Any revision surgeries or complications were also documented. This study was approved by our institutional review board.

### Indications for surgery

All patients received a diagnosis of unicompartmental osteoarthritis/osteonecrosis of the knee based on their medical history, physical examination, and radiographic evaluation [21]. Inclusion criteria to undergoing UKA were as follows: unicompartmental osteoarthritis/osteonecrosis of the knee, correctable varus/valgus deformity; flexion contracture ≤ 5°; intact cruciate ligaments. A cemented medial or lateral GKS ONE prosthesis (Permedica, Merate, Italy) was implanted by the senior surgeon (M.S.) in all patients. The procedure was performed without a tourniquet, using an 8–10 cm minimally invasive lateral or medial parapatellar approach.

### Indication of fast-track UKA and telerehabilitation protocol

Inclusion criteria for the fast-track UKA and telerehabilitation protocol were: body mass index (BMI) < 30 kg/m^2^, American Society of Anesthesiologists (ASA) class 1–2 [22], physical and psychological motivation, a supportive home environment, motivated and available caregiver(s), ability to use crutches, aptitude to manage the telerehabilitation digital program, a home internet connection, willingness to engage in early home rehabilitation followed by outpatient rehabilitation, and residence within 30 km of the hospital. Exclusion criteria were allergy/hypersensitivity to any of the drugs used in the fast-track protocol (cefazolin, hyperbaric bupivacaine, tranexamic acid, paracetamol, levobupivacaine, oxycodone, naloxone, ketorolac, tramadol, alizapride, parnaparin), congenital/acquired coagulopathy, active intravascular coagulation, acute occlusive vasculopathy, chronic cardiopathy, chronic use of oral anticoagulants/corticosteroids, malignancy, autoimmune disease, major bone defects requiring bone grafting, and refusal to sign the informed consent form.

#### Fast-track and telerehabilitation protocol

**Fig 2.**
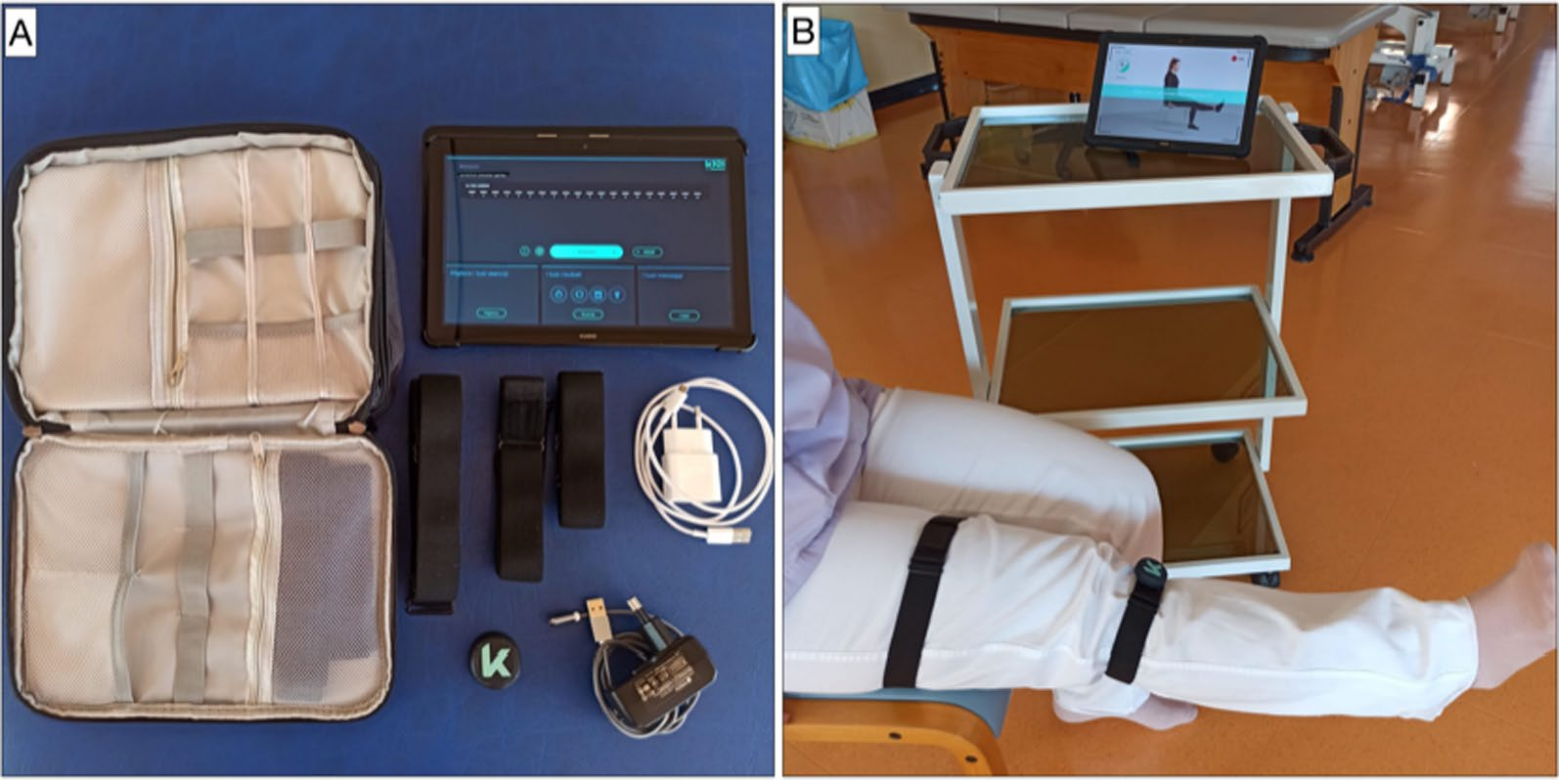
**a** KARI system home kit; **b** KARI system, sensor positioning

- Preoperative phase
  Pre-admission included medical, pre-anesthetic, and physiotherapy evaluation and assessment of family members, to assign roles to those who will be involved in patient care at home.
  Since a bladder catheter is not used, intraoperative water overload was avoided. Fats and meat were allowed up to 8 h before surgery, a light meal was allowed up to 6 h, and only fluids were allowed up to 2 h before surgery. Clear liquids (water, tea, chamomile tea) were permitted 2 h after surgery.
- Operative phase
  Patients received a 2 ml subarachnoid injection of 0.5% hyperbaric bupivacaine and breathed spontaneously with 2 l/min of supplementary oxygen. Normothermia > 36 °C was maintained with hot air. A minimally invasive procedure was used. Twenty minutes before the incision, patients received 1 g intravenous tranexamic acid [15, 23, 24]. Operative times were kept as short as possible (< 60 min) to reduce surgical stress [23, 25–27]. Before wound closure, local infiltration of anesthetic (LIA; 20 ml 0.25% levobupivacaine) [26, 28] was performed by the surgeon using a systematic technique. To ensure uniform anesthetic delivery to all tissues that had been incised, handled, or instrumented, 10 ml was injected into the anterior joint capsule and 10 ml into subcutaneous tissue [28, 29]. A standardized analgesia protocol with 1 g intravenous paracetamol three times a day was started 30 min before the end of the procedure.
- Postoperative phase
  Immediate full weight-bearing was initiated. X-rays were taken and examined within 3 h of the patient leaving the operating room, to enable early rehabilitation.
  Pain medication followed a standardized protocol based on the numerical rating scale (NRS). Four hours after surgery, patients received oral oxycodone/naloxone (5/2.5 mg tablets) every hour; over the next 72 h they received a 10/5 mg tablet twice a day, to ensure an NRS ≤ 4 [30]. Pain NRS > 4 was managed by ketorolac 30 mg/tramadol 100 mg, one vial in 100 ml saline, twice a day for 48 h.
- Hospital rehabilitation
  Four hours after the procedure, patients were examined and their rehabilitation chart was prepared by the physiotherapist. Active and passive limb mobilization was begun with the patient wearing elastic stockings on both lower extremities. Afterward, the patient was helped to take a short walk with the aid of a walker or crutches.
  Postoperative day (POD) 1 involved two physiotherapy sessions, again consisting of active and passive mobilization and of walking with crutches or a walker. On POD 2, the two sessions also involved negotiating stairs.
  Patients were usually discharged on POD 3. The discharge was agreed among the orthopedist, physiotherapist, internal medicine specialist, and patient, based on clinical condition and achievement of the short-term rehabilitation objectives. Patients were discharged home, where they received integrated home care according to the local health service protocols. The discharge letter, written by the orthopedist and the attending physician, specified the treatment program and the follow-up schedule.
- Home rehabilitation
  The home rehabilitation goals were set by the physiotherapist. The daily program involved a 90-min morning session with the physical therapist and telerehabilitation (at least 30 min) in the afternoon.
  At the time of the first home session, the patient received a telerehabilitation kit, KARI (Euleria, Rovereto, Italy), containing the devices, which, through an internet connection, enable remote support and training supervision, including a magnetic bluetooth inertial sensor; magnetic charging cable; adjustable elastic velcro bands for the trunk, thigh, tibia, and foot; a carrying bag; and a tablet with the app already installed (Fig. 2).

After 8–10 days, patients began an outpatient rehabilitation regimen (three times a week, 1 h a day).

### Standard pathway

The patients undergoing standard UKA and rehabilitation received routine surgical treatment and care. Rehabilitation began on POD 2 and involved a 90-min daily session with the physiotherapist in the morning and a 90-min session without the physiotherapist in the afternoon. After discharge, typically on POD 10–16, they began an outpatient rehabilitation program (1 h, three times a week) the duration of which was decided by the physiotherapist (usually 30–40 days). Compared with G1, in the standard protocol, patients LIA or analgesia protocol intraoperative were not use. No early mobilization and rehabilitation were begun immediately after surgical procedure. Painkillers were administered on demand.

### Clinical outcomes

Preoperative and postoperative functional outcomes were obtained retrospectively. The collected measures were as follows: WOMAC osteoarthritis index preoperative, on POD 2, 15, and 40, and at 12 months; ROM immediately after surgery, on POD 2, 15, and 40, and at 12 months. In addition, patient satisfaction was collected on the day 40 (3: very satisfied; 2: satisfied; 1: not completely satisfied; and 0: dissatisfied). Outcome results were obtained through clinical appointment.

### Statistical analysis

All analysis were conducted using Microsoft Excel (Microsoft) with the XLSTAT resource pack (XLSTAT-Premium, NY, USA). To adjust for differences in baseline characteristics, a propensity score matching (PSM) analysis was performed with 1:3 ratio (G1:G2).

To perform the matching operation, an optimal algorithm was used. In this way, it was possible to match each participant of the treatment group to three participants of the control group. Patients were eligible for matching if the difference of the estimated propensity score between G1 and G2 was within the caliper radius of 0.01* sigma. The strength of the association was estimated with the 95% confidence interval. Patients were matched for age, sex, body mass index (BMI), and laterality. Nonparametric (Mann–Whitney *U*) tests were used to assess continuous variables for significant differences between the two groups. Regarding categorical data, all measurements were compared using Fisher’s exact test. A *p*-value < 0.05 was defined as significant.

## Results

### Clinical outcomes

G1 demonstrated significantly better clinical outcomes in WOMAC scores on POD 2, 15, and 40 (*p* < 0.001, *p* < 0.001, *p* < 0.020, respectively) and ROM immediately after surgery, on POD 2, 15, and 40, and at 12 months (*p* < 0.001, *p* < 0.001, *p* = 0.014, *p* < 0.001, *p* = 0.003, respectively) (Table 2).

**Table 2:** Matched patient outcomes scores:

	Pre match			Post match		
Variable	Fast-track + telerehabilitation protocol	Standard protocol	*p*-Value	Fast-track + telerehabilitation protocol	Standard protocol	*p*-Value
WOMAC index score						
Preoperative, mean (SD) [range]	44.7 (13.1) [25.0–66.0]	57.1 (5.9) [45.0–68.0]	0.014	47.1 (12.1) [25.0–66.0]	54.4 (5.9) [45.0–64.0]	0.095
POD 2, mean (SD) [range]	66.1 (7.7) [56.0–79.0]	88.0 (4.7) [78.0–96.0]	< 0.001	70.0 (6.6) [63.0–79.0]	87.5 (4.9) [78.0–94.0]	< 0.001
POD 15, mean (SD) [range]	48.4 (6.1) [38.0–59.0]	65.9 (6.0) [57.0–78.0]	< 0.001	51.0 (5.8) [46.0–59.0]	65.3 (5.9) [57.0–78.0]	< 0.001
POD 40, mean (SD) [range]	26.8 (2.0) [24.0–30.0]	29.0 (2.2) [24.0–35.0]	< 0.001	26.4 (2.4) [24.0–30.0]	29.3 (2.0) [27.0–33.0]	0.020
12 months, mean (SD) [range]	25.5 (1.7) [24.0–28.0]	26.1 (1.5) [24.0–28.0]	0.153	25.1 (1.6) [24.0–28.0]	26.3 (1.5) [24.0–28.0]	0.112
ROM (°)						
Immediately after surgery, mean (SD) [range]	78.1 (10.9) [50.0–100.0]	40.2 (13.3) [10.0–70.0]	< 0.001	75.7 (6.1) [65.0–80.0]	40.2 (13.9) [10.0–65.0]	< 0.001
POD 2, mean (SD) [range]	90.3 (10.1) [80.0–110.0]	51.4 (14.4) [20.0–80.0]	< 0.001	85.0 (4.1) [80.0–90.0]	51.7 (15.2) [20.0–75.0]	< 0.001
POD 15, mean (SD) [range]	101.9 (10.0) [90.0–120.0]	91.3 (5.6) [80.0–100.0]	< 0.001	97.1 (4.9) [90.0–105.0]	90.0 (5.9) [80.0–100.0]	0.014
POD 40, mean (SD) [range]	129.4 (5.1) [120.0–135.0]	110.6 (6.2) [90.0–120.0]	< 0.001	126.4 (5.6) [120.0–135.0]	109.5 (6.3) [100.0–120.0]	< 0.001
12 months, mean (SD) [range]	128.1 (6.2) [110.0–135.0]	115.3 (7.6) [100.0–130.0]	< 0.001	127.9 (4.9) [120.0–135.0]	116.4 (8.2) [100.0–130.0]	0.003
Satisfaction questionnaire						
Very satisfied (%)	15 (83.3)	56 (71.8)	0.712	5 (71.4)	15 (71.4)	1.000
Satisfied (%)	3 (16.7)	17 (21.8)		2 (28.6)	5 (23.8)	
Not completely satisfied (%)	0 (0)	5 (6.4)		0 (0)	1 (4.8)	

*UKA*, unicompartmental knee arthroplasty; *WOMAC*, Western Ontario and McMaster University (WOMAC) osteoarthritis index; *SD*, standard deviation; *POD*, postoperative day; *ROM*, range of motion

Regarding patient satisfaction, no significant differences were found (*p* = 1.000).

### Complications and revisions

No complications occurred in either Group 1 or Group 2. In addition, none of the included patients required a revision surgery.

## Discussion

In this pilot clinical trial, the most important finding was that G1 patients experienced higher functional outcomes than G2 patients, with a better WOMAC score at POD 2, 15, and 40. Moreover, G1 patients achieved more satisfactory results in ROM at POD 2, 15, and 40, and 1-year follow-up compared with G2. After PSM, there was a marked improvement in WOMAC from POD 2 to 40 in patients in the G1 group. This difference, however, tended to disappear in the following days and months. Moreover, we noted a statistically significant difference in ROM from the immediate postoperative period up to month 12 (Table 2). Patients who underwent a UKA with a fast-track and telerehabilitation protocol could achieve better range of motion.

With regards to patient satisfaction, after PSM, there was no statistically significant difference between the G1 and G2 patients. We also noted that in G2, there was only one patient who reported that he was not completely satisfied with the intra/postoperative management (Table 2).

The LOS after knee and hip arthroplasty has been declining for some years, especially with fast-track protocols [15]. A large body of literature has highlighted some major advantages of such protocols, including fewer cardiopulmonary complications, shorter heparin prophylaxis, healthcare savings, and a lower risk of postoperative delirium, especially in older patients [31, 32]. Telemedicine affords quantitatively greater and more consistent patient monitoring, it is associated with greater compliance, thus earlier recovery, and ensures greater patient satisfaction while affording substantial hospital savings [33–35]. Several studies have found that in various medical branches, virtual rehabilitation is equivalent to conventional rehabilitation [36], for instance, some investigations of knee arthroplasty have found comparable results in terms of ROM, strength, stability, pain, and quality of life [19, 37]. The success of telerehabilitation depends on several factors, including patient physical and emotional aptitude, a suitable home environment, and especially, the ability to use electronic devices [38]. Indeed, poor familiarity with electronic tools often excludes older patients from these programs. Telerehabilitation may encourage more patients to choose a fast-track protocol. Critically, at a time when COVID-19 is still a cause for deep concern, fast-track procedures and telerehabilitation provide unique social distancing opportunities. Greater patient compliance is another key benefit of these programs.

Effective pain relief is crucial for early mobilization and rehabilitation; moreover, pain delays discharge [39, 40]. Pain management involves both anesthesia and analgesia. While all patients received subarachnoid spinal anesthesia, LIA could have helped the early mobilization in Group 1 [29]. Whereas patients managed by conventional rehabilitation tend to avoid movement, those in a fast-track regimen immediately begin passive and active mobilization. Early mobilization is also the main factor that helps reduce complications such as thromboembolic episodes [41]. A large study comparing a fast-track hip and knee arthroplasty protocol to the standard approach has described a clear reduction in complications such as cardiac ischemic events, mortality, and red blood cell transfusions [15]. The significantly greater knee mobility achieved by Group 1 patients at each assessment suggests that early mobilization is the chief factor affecting postoperative ROM. This suggests a key role for the fast-track surgery and rehabilitation program.

This study has several limitations. First, the sample size is relatively small (*n* = 28) to detect the treatment effect of the fast-track and telerehabilitation protocol. Second, this is a nonrandomized study and includes a retrospective design. Third, although the propensity score analysis was used, comorbidities were not matched on, and this may have influenced our results. Fourth, the different protocol of pain management between groups, which would affect the early function (i.e., LIA).

## Conclusions

In this pilot study of 28 subjects, the fast-track and telerehabilitation protocol in UKA proved to be safe and effective. At 12-months follow-up, both groups reported favorable outcomes after UKA. However, the G1 patients showed encouraging results regarding WOMAC and ROM compared with propensity score-matched patients in G2. No differences were found in grade of satisfaction and postoperative complications rate. Future studies with a larger population are warranted to explore the effects of fast-track and telerehabilitation protocols.

## Data Availability

The datasets generated and/or analyzed during the current study are not publicly available, but they are available from the corresponding author on reasonable request.

## Abbreviations

UKA: Unicompartmental knee arthroplasty (UKA)
G1: Group 1
G2: Group 2
WOMAC: Western Ontario and McMaster University
ROM: Range of motion
LOS: Length of stay
BMI: Body mass index
ASA: American Society of Anesthesiologists
PSM: Propensity score match
LIA: Local infiltration of anesthetic
NRS: Numerical rating scale
POD: Postoperative day
SD: Standard deviation
